# Influence of air supply on coal spontaneous combustion during support withdrawal in fully mechanized coal mining and its prevention

**DOI:** 10.1038/s41598-021-98422-w

**Published:** 2021-09-29

**Authors:** Xiaowen Zhang, Xihua Zhou, Gang Bai, Cheng Wang

**Affiliations:** 1 College of Safety Science & Engineering, Liaoning Technical University, Fuxin, 123000 Liaoning China; 2 Key Laboratory of Mine Thermodynamic Disasters & Control of Ministry of Education, Fuxin, 123000 Liaoning China; 3 Guoneng Ningxia Coal Mine Group Co., Ltd. Yangchangwan Coal Mine, Yinchuan, 751409 Ningxia China

**Keywords:** Energy science and technology, Engineering

## Abstract

It is necessary to change the air supply rate of the working face during the withdrawal of fully mechanized mining, making it important to study the oxidation characteristics of coal samples under different air supply rates. Through a self-made temperature-programmed experimental device, our focus was on studying the change laws of indicator gases released during the low-temperature (303.15–473.15 K) oxidation stage when the air supply rates of the coal samples were 0.67, 1.33, 2, 2.67, and 3.33 mL/s. The experimental results showed that the air supply increased, the concentrations of CO, C_2_H_6_, C_3_H_8_, C_2_H_4_, and C_2_H_2_ generated by the coal sample at the same temperature decreased, and the oxidation process decelerated. The initial temperatures of the four hydrocarbon gases were delayed to varying degrees with the increase in the air volume, and C_2_H_4_ was found to be more suitable as a hydrocarbon gas for the early warning of coal spontaneous combustion. Surface fitting was applied to analyze the change law of the CO generation rate under the combined effect of temperature and air supply; the change was divided into three stages. The CO concentration model at the upper corner of the working face during the withdrawal period was deduced, and comprehensive safety measures were put forward to prevent coal spontaneous combustion during the withdrawal period.

## Introduction

The spontaneous combustion of coal is detrimental to the safe operation of coal mines. This phenomenon not only causes wastage of resources and equipment damage, but can also lead to gas and coal dust explosions, induce secondary disasters, and cause serious casualties and property losses^1–3^. The goaf area is most prone to spontaneous combustion^4^. Particularly when mining is stopped during the support withdrawal period, a reduction in air supply exacerbates the heat dissipation condition behind the hydraulic support, easily leading to heat accumulation, which is a fundamental cause of fires in fully mechanized caving faces^5^. Because of the long working face and the large number of supports, the withdrawal period is too long. This provides sufficient time for the coal that remains in the goaf to fully oxidize and undergo spontaneous combustion. Therefore, an early prediction of spontaneous combustion during the withdrawal period, analysis of the influence of air supply on the combustion process, and effective measures are of great significance to the prevention and control of fires in coal mines.

Currently, the most effective and widely used measure to predict and prevent spontaneous combustions in the goaf is to predict the temperature of the goaf and the index gases accompanying the spontaneous combustion^6,7^. The combustion of coal is accompanied by the release of gases under the conditions of heat storage oxidation and high temperatures. The temperature-programmed experiment is an effective method to study the spontaneous combustion characteristics of coal. Related studies on the thermal oxidation of coal have shown that the concentration of the combustion products increases with the increase in the coal temperature^8–10^. Wu^11^ and Dong et al.^12^ found that as the oxidation temperature of the coal samples increases in the experiment, the CO concentration exhibits an exponential increase trend with the increase in the coal temperature. When the coal temperature exceeds a certain critical temperature, the gas concentration rises rapidly.

Scholars have conducted related research on the influence of air supply on the spontaneous combustion of coal. Kim^13^, Walters^14^, and Mastalerz et al^15^. believed that changes in the air velocity have a greater impact on the heat transfer of coal and that external oxygen supply conditions have a greater impact on spontaneous combustion; however, this effect is relatively weak when the air velocity is low. Chamberlain et al.^16^ and Yuan and Smith^17^ studied the effects of oxygen concentration in the air and the air supply rate on the spontaneous combustion of coal. They showed that under an initial temperature of 343.15 K, changing the ventilation rate has no evident effect on CO production. Phillips et al.^18^ and Rambha and Ren^19^ obtained the gas flow when the dry coal sample had the maximum oxidation potential. Więckowski et al.^20^ showed that with the increase in the air flow, the ratio of CO/CO_2_ produced by the spontaneous combustion of coal samples increases with temperature and that the flow rate has an impact on the index value.

However, most of the above-mentioned studies were on the influence of air volume on the concentration of the spontaneous combustion products and oxygen consumption, and a comprehensive analysis of the influence of air volume on the index of the CO generation rate is lacking.

In this work, experimental coal samples were obtained from the Yangchangwan Coal Mine, which belongs to the Eastern Ningxia Mining Area, a billion-ton coal base in China, with low metamorphism lignite that is prone to spontaneous combustion^21,22^. In the past, spontaneous combustion in the goaf of this mine has occurred while mining was stopped and while removing the support. To avoid such combustion, this study developed a temperature-programmed experimental device to oxidize the coal sample and analyze the influence of the gas product concentration on the temperature under different air volume rates. Based on a prediction model for the upper-corner CO concentration combined with the actual site parameters, this paper summarizes effective measures that can be taken to prevent spontaneous combustion during the withdrawal period.

## Analysis of CO concentration at upper corner when coal mining is halted

The source of CO at the corners of the fully mechanized mining face is analyzed. Most of the CO comes from the oxidation of leftover coal in the goaf, while a small amount comes from the release of broken coal bodies due to the advance of the working face, blasting, and influx of the adjacent working areas^23^. Therefore, the CO concentration at the upper corner can be expressed as:

1 $$C_{co} = \left( {W_{1} + W_{2} } \right)/Q_{L}$$

where ${C}_{co}$ is the CO concentration at the upper corner of the working face (mol/m^3^),${Q}_{L}$ is the air leakage volume in the goaf (m^3^/s), ${W}_{1}$ is the amount of CO released by the oxidation of the residual coal in the goaf per unit time (mol/s), and ${W}_{2}$ is the amount of CO produced by other factors (mol/s). When the working face is no longer advancing, the amount of ${W}_{2}$ can be ignored. According to the theory of spontaneous combustion in the three zones of the goaf^24,25^, the amount of CO produced by the oxidation of the residual coal in the goaf ${W}_{1}$ is expressed as^26^:

2 $$W_{1} = 10^{6} \left( {\alpha X_{1} + \beta X_{2} } \right)S\left( {1 - \eta } \right)\varphi_{CO}$$

where $\alpha$ is the oxidation correction coefficient of the leftover coal in the heat dissipation zone, which is typically in the range of 0.3–0.5 for fully mechanized mining faces; ${X}_{1}$ is the width of the heat dissipation zone, m; *β* is the oxidation correction coefficient of the leftover coal in the oxidation zone, which is in the range of 0.8–1.0 under normal air leakage conditions (when the air leakage rate is < 1%, *β* is < 0.5); ${X}_{2}$ is the width of the oxidation zone, in m; *S* is the cross-sectional area of the coal seam in the working face, m^2^; $\eta$ is the recovery rate of the coal seam in the working face, %. ${\varphi }_{CO}$ is the release rate of CO, mol/(cm^3^·s). It can be measured from the coal oxidation temperature experiment and expressed as^4^:

3 $$\varphi_{CO} = \frac{{C_{CO}^{2} q\ln a}}{{AL\left[ {1 - a^{{ - \frac{1}{a}}} } \right]}}$$

where *a* is the ratio of the oxygen concentration at the entrance ${C}_{{O}_{2}}^{1}$ to the oxygen concentration at the outlet ${C}_{{O}_{2}}^{2}$;${C}_{CO}^{2}$ is the outlet CO concentration, mol/cm^3^; *A* is the cross-sectional area of the coal sample tank, cm^2^; *q* is the air supply rate of the experimental system, mL/s; *L* is the height of the coal in the coal sample tank, cm.

The expression for the CO concentration at the upper corner of the working surface when mining is halted and supports are being withdrawn is:

4 $$C_{co} = 10^{6} \left( {\alpha X_{1} + \beta X_{2} } \right)S\left( {1 - \eta } \right)\varphi_{CO} /Q_{L}$$

Combined with the actual parameters of the working face at the site^27^, the predicted value of the CO concentration at the upper corner corresponding to different characteristic temperatures during the withdrawal period of fully mechanized mining can be derived. Therefore, a coal temperature program test was implemented to analyze the gas release law of the coal samples at different characteristic temperatures.

## Coal oxidation temperature-programmed experiment

### Experimental system and method

**Figure 1:**
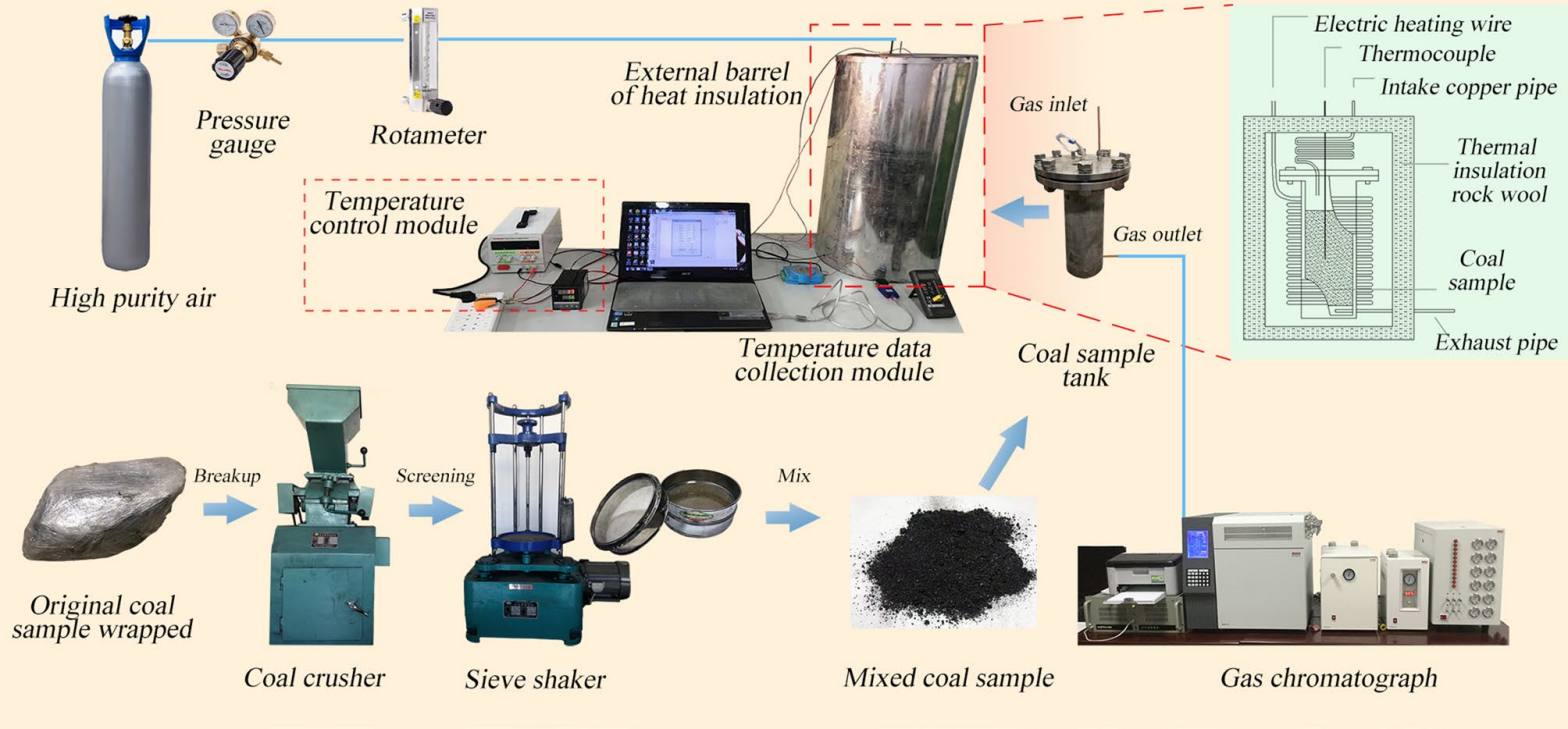
Temperature-programmed experimental system for coal oxidation.

To analyze the influence of air supply on the process of coal carbon oxidation, an oxidation temperature-programmed chromatographic analysis test was conducted using the developed temperature-programmed experimental device. Figure 1 shows the experimental system comprising four parts: gas circuit, coal sample, heating temperature control, and data collection. The experimental coal samples were obtained from the 20,606 working face of Yangchangwan Coal Mine. After being packaged and transported to a laboratory peeling surface, they were crushed using a jaw crusher. The samples were then sieved to sizes of 0–0.5, 0.5–0.9, 0.9–3, 3–5, and 5–8 mm using a vibrating sieve machine, and a total of 1 kg was mixed based on the mass ratio and loaded into the coal sample tank which has a diameter of 9.2 cm and a height of 25 cm.

The coal sample tank was placed in an insulating sleeve, and the heating tube in the cylinder surrounded the coal sample tank body for heating. The rock wool was attached to the inner wall of the sleeve to reduce the heat exchange between the internal heating tube and the external environment. The coiled arrangement of the intake copper pipe on the outside of the coal sample tank ensured that the intake air fully preheated the upper end of the coal sample tank and the lower end exhausted to the external chromatograph. The heating tube was controlled using an external XMTD temperature control module to control the heating temperature and heating rate. A K-type armored thermocouple was inserted in the tank to monitor the temperature of the coal sample, and the software recorded the temperature data in real time.

From the similarity theory, the temperature-programmed experiment can produce a law similar to that of the actual coal spontaneous combustion point gas release in the goaf^28^ before the experiment, ventilate the air, and check the air tightness of the device. According to literature^29^, the upper and lower limits of the air supply flow rate for the oxidation temperature test should be set to 0.67 and 3.33 mL/s, respectively. At the beginning of the experiment, the pressure reducing valve of the high-purity air cylinder was opened, and the air pressure was adjusted to 0.1 MPa. The flow rate of the rotameter in each experiment was set to 0.67, 1.33, 2, 2.67, and 3.33 mL/s. The heating rate of the heating device was set to 1 K per min. After the air was heated and reacted with the coal sample through the coal sample tank, the gas sample was collected through the drying tube and placed in a GC4000A chromatograph for analysis. The gas products were recorded once every at 5 K from 303.15 to 473.15 K and every 50 K from 473.15 to 873.15 K. Chromatographic TCD and FID were used for detection. Nitrogen and hydrogen were introduced as the carrier gases. The flow rate of both the carrier gas was 0.5 mL/s. The bridge temperature of the TCD was 393.15 K, and the detector temperature was 373.15 K. The result analysis was performed after the system stabilized. After the experiment was repeated five times, the data were averaged for the result analysis.

### Experimental results

#### CO concentration

**Figure 2:**
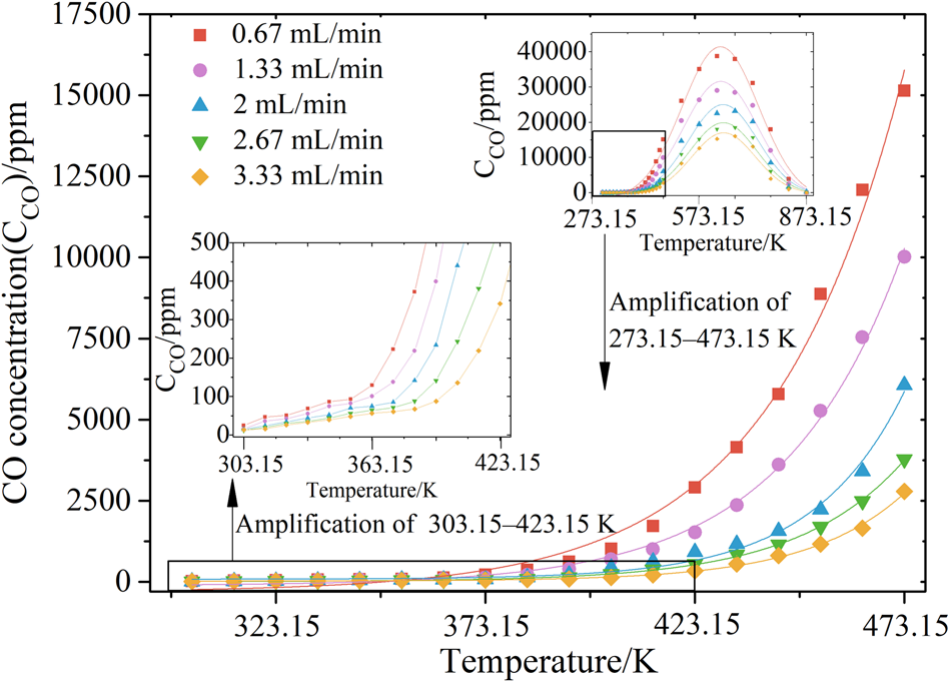
Variation curve of the CO concentration in the spontaneous combustion process of coal samples under different air volume rates.

From the result, the CO volume concentration data generated by each coal sample during the spontaneous combustion process were curve-fitted, and the CO concentration released by the coal samples under different air supply conditions was obtained, as shown in Fig. 2. The figure shows that the entire oxidation process exhibits a Gaussian function distribution under different air supply conditions.

For the early warning of coal spontaneous combustion, the focus should be on the analysis in the low-temperature (303.15–473.15 K) oxidation stage. In this stage, the distribution of the CO concentration ${C}_{CO}$ can be fitted using an exponential function ${C}_{CO}=a+b{e}^{-T/c}$ with temperature *T*, where ${C}_{CO}$ is in ppm, and *T* is in K. Table 1 lists the parameter values. From the concentration change curve, the low-temperature oxidation and heating process of the coal sample can be divided into two stages: a stable heat storage stage and an accelerated oxidation stage. The critical temperature of CO release is typically used to divide the two stages^30^. Figure 2 shows a comparison of the CO concentration release laws of the same coal sample under air volume rates of 0.67, 1.33, 2, 2.67, and 3.33 mL/s. CO gas can be detected at a low temperature of 30 °C, and it is inferred that a part of the CO gas adsorbed inside the coal body is released. It is verified that even a small amount of CO gas can be detected under normal conditions where spontaneous combustion does not occur at the site^16,31^. When the air supply rate is set to 0.67, 2, and 3.33 mL/s, the CO concentration released by the coal sample increases significantly after 363.15, 373.15, and 393.15 K, respectively. After this temperature, the rate of increase in the CO concentration increases sharply, and it develops toward deep oxidation.

**Table 1:** Curve parameters of CO concentration varying with temperature under an air volume rate range of 0.67–3.33 mL/s.

Parameters	Air volume rate (mL/s)				
	0.67	1.33	2	2.67	3.33
a	− 308.37	− 112.89	83.94	12.65	24.55
b	4.84e−3	7.12e−4	2.12e−6	2.93e−5	3.11e−6
c	− 31.51	− 28.68	− 21.78	− 25.35	− 22.99

At room temperature, coal not only physically and chemically adsorbs oxygen in the air, but also desorbs a small amount of CO adsorbed and attached onto the micropores in the coal^32,33^. At the same time, a part of the active structure of coal molecules undergoes a multistep reaction of coal–oxygen recombination to generate CO and other gases^34^. This process is dominated by the exothermic effect as a whole, which causes the temperature of the coal body to rise. As the oxidation process progresses, the coal temperature continues to rise, and the physical adsorption and desorption processes between coal and oxygen tend to be balanced, and chemical adsorption is the dominant mechanism. The speed gradually increases, and the heat release intensity also gradually strengthens. When the coal temperature rises to the critical temperature, chemical adsorption and desorption tend to balance again. From a macroscopic viewpoint, the rate of coal consumption of oxygen increases, and the production of the reaction product CO begins to increase^35^. In the experiment, increasing the air supply volume takes away the heat of the coal body and slows the rising process of the coal temperature, and the critical temperature rises accordingly.

#### Hydrocarbon gas concentration.

**Figure 3:**
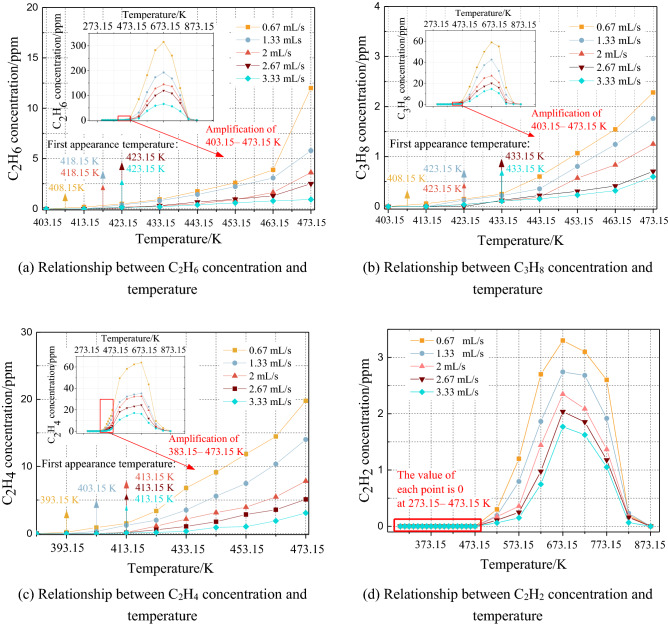
Relationship between gas concentration and temperature under an air volume rate range of 0.67–3.33 mL/s.

**Figure 4:**
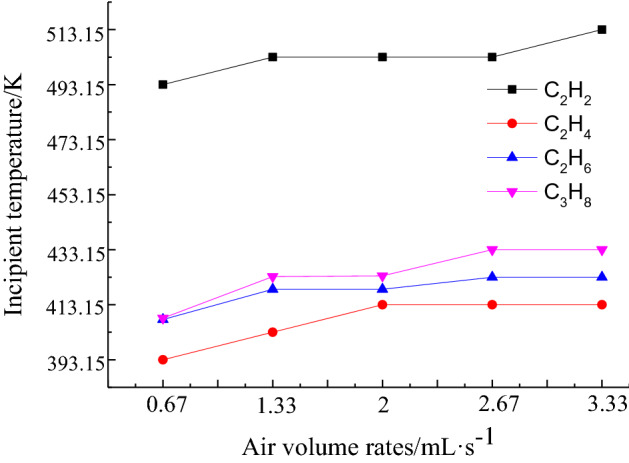
Variation in the first appearance temperatures of C_2_H_6_, C_3_H_8_, C_2_H_4_, and C_2_H_2_ under different air volume rates.

Four types of typical hydrocarbon gases were selected: saturated hydrocarbon C_2_H_6_, C_3_H_8_, unsaturated hydrocarbon C_2_H_4_, and C_2_H_2_. An analysis of the concentration test results shows that the gas concentration changes with the coal temperature under different air supply conditions, as shown in Fig. 3. The release concentrations of the four hydrocarbon gases decrease with increasing air volume rate. Figure 4 shows the relationship between the initial temperature of the four hydrocarbon gases and the air volume rate.

The initial temperatures of C_2_H_6_ and C_3_H_8_ under different air volume rates are relatively close, at 408.15 K, under a minimum air supply of 0.67 mL/s. When the air supply is increased to a maximum of 3.33 mL/s, the initial temperatures of C_2_H_6_ and C_3_H_8_ increase to 423.15 K and 433.15 K, respectively.

In the low-temperature oxidation stage, the critical temperature for the accelerated release of the C_2_H_6_ and C_3_H_8_ gases under different air volume rates is in the range of 453.15–473.15 K. Above this temperature, the coal oxidation reaction intensifies, and the concentrations of the C_2_H_6_ and C_3_H_8_ gases produced increase sharply. Above 673.15 K, the C_2_H_6_ and C_3_H_8_ gas produced by the coal drops sharply after reaching the peak value. At this stage, the combustion state of the coal sample begins to weaken.

Figure 3c shows that the initial temperature of C_2_H_4_ is the lowest, 393.15 K at 0.67 mL/s. When the air volume rate increases to 2 mL/s, the initial temperature rises to 413.15 K. The initial temperature of C_2_H_4_ remains unchanged at 413.15 K when the experimental air supply continues to increase. The initial temperature of the C_2_H_4_ gas under different air volume rates is the lowest, making it a suitable index gas to judge the combustion temperature status in advance.

Figure 3d shows that the concentration of the C_2_H_2_ gas produced is much lower than those of the other hydrocarbon gases. When C_2_H_2_ gas is detected, the coal temperature is over 493.15 K, making it unsuitable as an indicator gas for the early prevention of coal spontaneous combustion.

When the coal temperature exceeds the critical temperature, the coal–oxygen recombination will enter the automatic acceleration stage. When the coal temperature exceeds the second characteristic temperature, the side chains of the coal molecules break from the main structure and participate in the oxidation reaction. As the concentration of the active groups increases, the chances of collision between groups increase, and macroscopically, various hydrocarbon gaseous products are gradually generated^36^. In summary, at higher air volumes, the heat generated by coal combustion is dissipated more easily, and the coal–oxygen recombination reaction rate decreases. As a result, as the air volume rate increases at the same temperature, the release concentrations of the four hydrocarbon gases decrease. The initial temperatures of these gases lag due to the increase in the air volume rate.

#### CO generation rate

**Figure 5:**
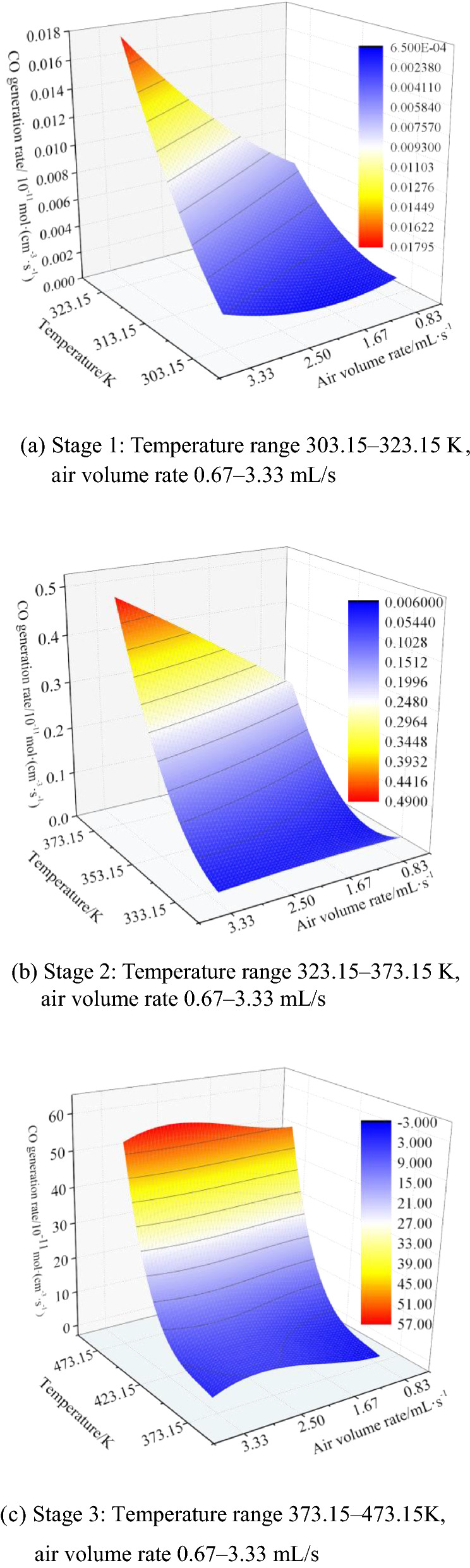
Variation in the CO production rate of the coal samples with temperature and air volume.

The measured CO generation rate varies with the temperature and air supply. The experimental results have the characteristics of stages. Based on the magnitude and change law of the CO generation rate, it can be divided into three stages. Figure 5 shows the 3D surface of the air volume rate and temperature on the combined effect of ${\varphi }_{CO}$.

The temperature range corresponding to the first stage is 303.15–323.15 K, as shown in Fig. 5a. The CO production rate in this stage shows a gradual upward trend with the increase in the temperature and air volume rate. At 0.67 mL/s, the CO production rate increases with temperature from 0.00106 × 10^−11^ mol/(cm^3^·s) to 0.00681 × 10^−11^ mol/(cm^3^·s). At 3.33 mL/s, the CO production rate increases with temperature from 0.00155 × 10^−11^ mol/(cm^3^·s) to 0.01674 × 10^−11^ mol/(cm^3^·s). Increasing the air supply will increase the CO generation rate at the same temperature. In this stage, the CO production rate is low and unstable. The main source of CO production is that coal molecules produce a large number of bond breaks due to external forces, which react with oxygen. The regular change in the CO gas produced obeys a binary quadratic polynomial distribution. At this stage, the quantitative relationship equation for the CO production rate ${\varphi }_{CO}$, temperature *T*, and air supply *Q* can be fitted as follows: ${\varphi }_{CO}$ = 1.32–8.57e-3* T*-6.11e-2*Q* + 1.39e-5*T*^2^ + 5.19e-4*Q*^2^ + 1.95e-4*TQ*.

The temperature in the second stage corresponding to the relationship between the CO generation rate and the combined effect of the air volume rate and temperature is between 323.15 and 373.15 K, and the rate of change in the CO generation rate in this stage is significantly higher than that in the first stage. The influence law of the temperature and air volume rate on the generation rate is consistent and positively correlated with the first stage. When the air volume rate is 0.67 mL/s, the CO generation rate increases with the temperature, from 0.0082 × 10^−11^ mol/(cm^3^·s) to 0.22683 × 10^−11^ mol/(cm^3^·s). At 3.33 mL/s, the CO generation rate increases from 0.0266 × 10^−11^ mol/(cm^3^·s) to 0.48343 × 10^−11^ mol/(cm^3^·s). This shows that the source of CO production in this stage is affected by coal–oxygen recombination, exhibiting an accelerated reaction trend. This inference is mainly due to the cleavage of the surface functional groups of the coal molecules and the recombination of oxygen to produce CO gas. The entire curve change in this stage also obeys the binary quadratic polynomial equation, and the quantitative relationship equation can be fitted as: ${V}_{cO}$ = 22.41–1.31e-1* T*-6.94e-1*Q* + 1.92e-4*T*^2^-2.29e-3*Q*^2^ + 2.14e-3*TQ*.

The third stage of the change in the CO generation rate of the coal samples corresponds to a temperature between 373.15 and 473.15 K, as shown in Fig. 5c. Under the same temperature conditions, when the air volume rate increases from 0.67 mL/s to 2 mL/s, the CO generation rate increases with the increase in the air volume rate. When the air volume rate is increased from 2 mL/s to 2.67 mL/s, the CO generation rate remains largely unchanged. Under the same conditions of 473.15 K and air volume rates of 2, 2.67, and 3.33 mL/s, the ${\varphi }_{CO}$ values are 58.50042, 60.24115, and 54.45101 mol/(cm^3^·s), respectively. At this time, the air supply is increased, and the air leakage is close to the upper limit. A state of inhibited oxidation can be observed, which decreases the oxidation rate. This shows that the inhibitory effect of air supply on the CO generation rate is mainly reflected in the accelerated CO release stage. The data in this stage obey the binary cubic surface equation, which can be fitted as: ${\varphi }_{CO}$ =  − 4889.6 + 37.51* T*-9.61e-2*T*^2^ + 8.22e-5*T*^3^-7.58*Q* + 7.46*Q*^2^-1.53*Q*^3^.

## Application and countermeasures

### Overview of working face

The average coal seam thickness of the 20,606 working face is 3.48 m, and the design mining height is 2.9 m. A fully mechanized mining method is adopted, and the roof is managed using caving methods. The inclined length of the working face is 247.3 m, and the length of the working face can be 1570 m. A U-shaped full air pressure upward ventilation is adopted during the working face. The actual air distribution rate is approximately 14.95 m^3^/s, and the air leakage rate of the goaf is measured to be 0.53 m^3^/s.

### Three-zone observation and prediction calculation

**Figure 6:**
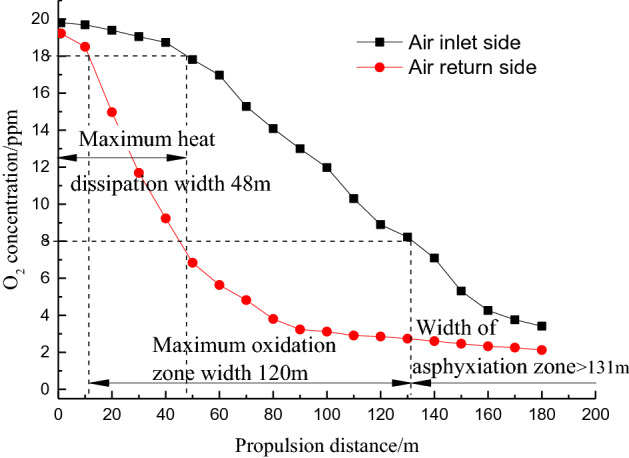
Three-zone division of the goaf in the 20,606 working face.

To study the distribution of the oxygen concentration in the goaf of the 20,606 working face, monitoring points were pre-arranged at the inlet and return air sides of the goaf in the initial stage of the working face. As the working face advances, a downhole special suction pump is used to pass through the embedded bundle pipe. The gas is collected, and the oxygen concentration at different distances from the measuring point in the goaf to the working surface is analyzed. Based on the judgment theory for areas prone to spontaneous combustion^4,25^, the oxygen volume fraction in the heat dissipation zone is greater than or equal to 18%; the oxygen volume fraction in the oxidation heating zone ranges from 18 to 8%; the oxygen volume fraction in the asphyxiation zone is < 8%. The measured O_2_ volume fraction varies with the advancing distance of the working surface, as shown in Fig. 6.

The measured oxygen concentration index at the 20,606 working face is divided into a “three zone” range of the goaf spontaneous combustion: the maximum width of the heat dissipation zone is 48 m on the inlet side; the width of the heat dissipation zone on the inlet side is 48–131 m, and the width of the oxidation zone is 48–131 m on the return side. The width of the oxidation zone is 11–45 m, and the maximum width of the oxidation zone is 120 m. Based on the actual measured oxygen concentration and air leakage conditions, the CO concentration at the upper corner of the working face calculated using Eq. (4) should be lower than 44.6 ppm for a coal temperature of 303.15 K when mining is stopped at the 20,606 working face. The lowest critical temperature of accelerated oxidation is 363.15 K. The upper-corner CO concentration should be lower than 873.5 ppm.

### On-site response measures

Coal spontaneous combustion should meet the following four conditions at the same time^37,38^: ① Coal is broken and accumulated and has a tendency to undergo spontaneous combustion ② continuous oxygen supply conditions ③ good heat storage environment ④ sufficient reaction time. Measures should also be taken to break the above conditions to prevent spontaneous combustion of the coal during the withdrawal of fully mechanized mining.

The experimental results of this study show that, in the low-temperature heat storage stage of coal spontaneous combustion, reducing the air volume rate can help reduce the rate of CO generation and decelerate the combustion process. The air volume supply can be reduced under the condition of meeting the minimum air volume of the working face, and a wind screen can be installed in the auxiliary lane. This can effectively hinder the supply of oxygen from the air inlet tunnel to the goaf.

When mining at the working face is close to the stop line, the working face should be recovered along the roof as much as possible to minimize the amount of floating coal in the goaf and reduce the risk of spontaneous combustion. This can minimize the top coal from falling in the goaf and leaving broken floating coal and at the same time reduce the space for oxygen flow. Sufficient time should be allowed for the later withdrawal of equipment at the working face. At this time, accelerating the progress of coal mining can reduce the reaction time of coal and oxygen in the goaf, thereby slowing down the spontaneous combustion of coal.

**Figure 7:**
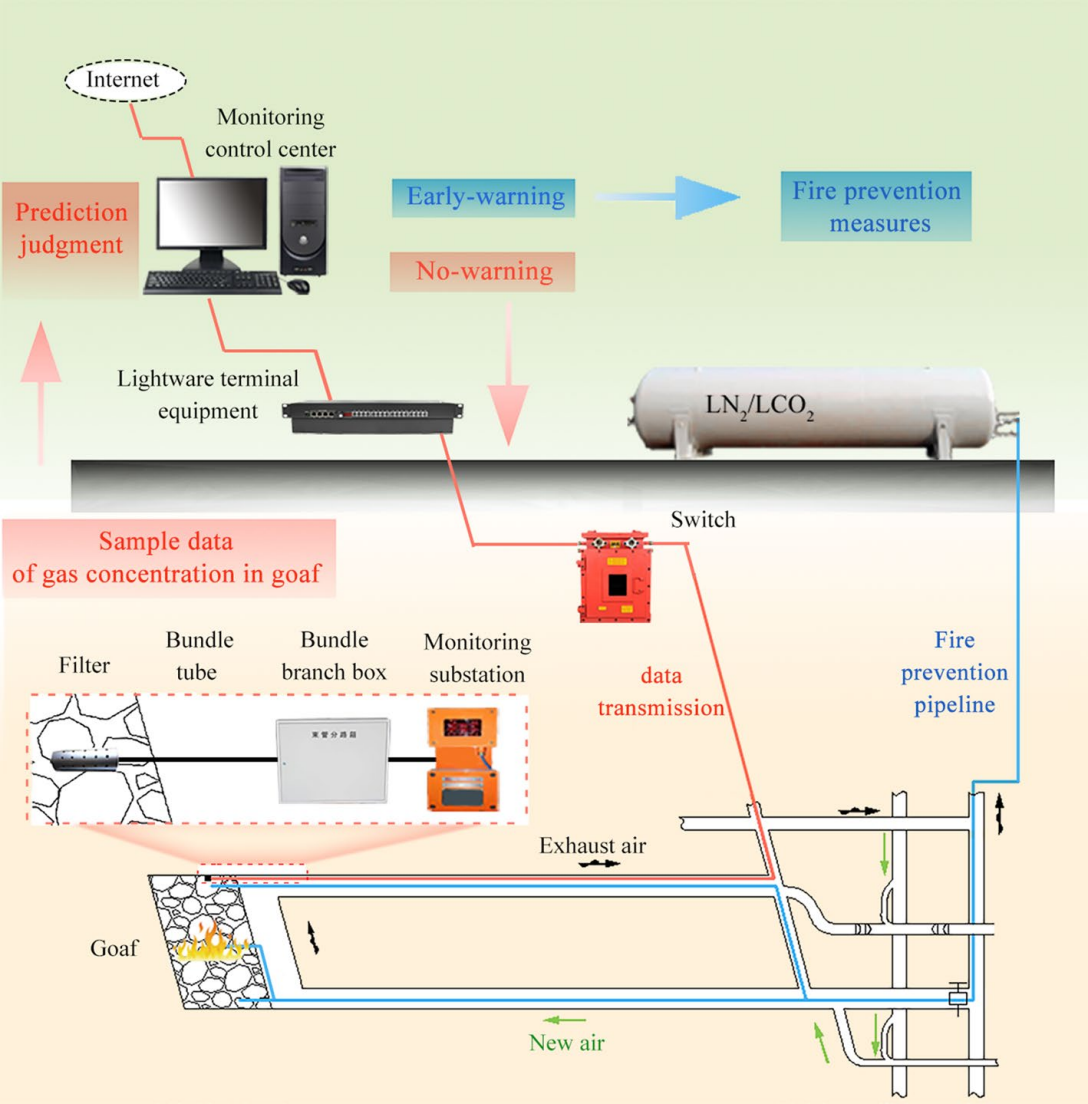
Monitoring linkage system for fire prevention and extinguishing at the working face.

A low-temperature inert liquid (LN_2_/LCO_2_) is applied to prevent the spontaneous combustion of coal. After the liquid is injected into the goaf, it rapidly absorbs heat and expands in volume, reducing the temperature of the remaining coal in the goaf and significantly reducing the oxygen concentration in the goaf environment^39–41^. The spontaneous combustion of coal through the simultaneous oxygen supply and heat storage conditions is prevented, and sufficient time is provided for the withdrawal of the rack. As shown in Fig. 7, inerting firefighting pipelines are pre-embedded from the final mining line to the goaf to prevent and control areas prone to spontaneous combustion during the withdrawal period. The inertia injection firefighting system is linked through the ground dispatch center. Using a laser beam tube monitoring system, the gas analysis and calibration can be controlled via a computer, and the analysis results can be transmitted to the ground through an optical fiber to realize the automation of the monitoring and analysis and display the real-time changes in the data. Underground real-time monitoring and monitoring data analysis has the highest efficiency for controlling any fire situations in the goaf and can help improve the safety of the final mining and reduce the need to take drastic prevention measures during the suspension and withdrawal period.

## Conclusions

This study conducted temperature-programmed experiments on coal samples collected from the Yangchangwan Coal Mine. The results showed that changing the air supply conditions has a significant impact on the development of coal spontaneous combustion. An analysis of the experimental results in the low-temperature (303.15–473.15 K) oxidation stage under different air volume rates showed that, under the same air supply conditions, the generated CO concentration increases with the increase in the coal temperature, following a power-exponential function. With the increase in the experimental air volume rate, the concentrations of the released hydrocarbon gases decreased, and their initial temperatures showed a hysteresis phenomenon. Under the influence of different air volume rates, the lowest initial temperature of C_2_H_4_ was found to be 393.15–413.15 K, and the highest initial temperature of the C_2_H_2_ gas was in the range of 493.15–513.15 K. The C_2_H_4_ gas was most suitable as the indicator gas for the early warning of coal spontaneous combustion.

The change law of the CO generation rate under the combined effect of air volume rate and temperature could be divided into three stages based on the change magnitude and law. The corresponding temperature ranges are 303.15–323.15, 323.15–373.15, and 373.15–473.15 K. In the first and second stages, the CO production rate showed a gradual upward trend with the increase in the temperature and air volume rate. In the third stage, the CO production rate increased with increasing temperature; however, at the same temperature, with the increase in the air volume, it first increased and then decreased. The inhibitory effect of increasing the air volume rate on CO release from coal oxidation is mainly reflected in the third stage of accelerated CO release.

The paper summarized comprehensive measures that can help prevent spontaneous combustion during the support withdrawal period, reduce the air leakage in the goaf during this period, reduce the amount of remnant coal, and enable real-time monitoring. Pre-arranged measures, such as a linkage system for firefighting, can help prevent/extinguish fires in a timely manner. Put together, these measures can ensure a smooth progress of the support withdrawal operation in coal mines.
